# Voluntary Running Aids to Maintain High Body Temperature in Rats Bred for High Aerobic Capacity

**DOI:** 10.3389/fphys.2016.00311

**Published:** 2016-07-25

**Authors:** Sira M. Karvinen, Mika Silvennoinen, Hongqiang Ma, Timo Törmäkangas, Timo Rantalainen, Rita Rinnankoski-Tuikka, Sanna Lensu, Lauren G. Koch, Steven L. Britton, Heikki Kainulainen

**Affiliations:** ^1^Department of Biology of Physical Activity, Neuromuscular Research Center, University of JyväskyläJyväskylä, Finland; ^2^Department of Health Sciences, University of JyväskyläJyväskylä, Finland; ^3^Gerontology Research Center and Department of Health Sciences, University of JyväskyläJyväskylä, Finland; ^4^Centre for Physical Activity and Nutrition Research, School of Exercise and Nutrition Sciences, Deakin UniversityMelbourne, VIC, Australia; ^5^Department of Anesthesiology, University of Michigan Medical SchoolAnn Arbor, MI, USA; ^6^Department of Molecular and Integrative Physiology, University of Michigan Medical SchoolAnn Arbor, MI, USA

**Keywords:** aerobic capacity, aging, body temperature, physical activity, skeletal muscle

## Abstract

The production of heat, i.e., thermogenesis, is a significant component of the metabolic rate, which in turn affects weight gain and health. Thermogenesis is linked to physical activity (PA) level. However, it is not known whether intrinsic exercise capacity, aging, and long-term voluntary running affect core body temperature. Here we use rat models selectively bred to differ in maximal treadmill endurance running capacity (Low capacity runners, LCR and High capacity Runners, HCR), that as adults are divergent for aerobic exercise capacity, aging, and metabolic disease risk to study the connection between PA and body temperature. Ten high capacity runner (HCR) and ten low capacity runner (LCR) female rats were studied between 9 and 21 months of age. Rectal body temperature of HCR and LCR rats was measured before and after 1-year voluntary running/control intervention to explore the effects of aging and PA. Also, we determined whether injected glucose and spontaneous activity affect the body temperature differently between LCR and HCR rats at 9 vs. 21 months of age. HCRs had on average 1.3°C higher body temperature than LCRs (*p* < 0.001). Aging decreased the body temperature level of HCRs to similar levels with LCRs. The opportunity to run voluntarily had a significant impact on the body temperature of HCRs (*p* < 0.001) allowing them to maintain body temperature at a similar level as when at younger age. Compared to LCRs, HCRs were spontaneously more active, had higher relative gastrocnemius muscle mass and higher UCP2, PGC-1α, cyt c, and OXPHOS levels in the skeletal muscle (*p* < 0.050). These results suggest that higher PA level together with greater relative muscle mass and higher mitochondrial content/function contribute to the accumulation of heat in the HCRs. Interestingly, neither aging nor voluntary training had a significant impact on core body temperature of LCRs. However, glucose injection resulted in a lowering of the body temperature of LCRs (*p* < 0.050), but not that of HCRs. In conclusion, rats born with high intrinsic capacity for aerobic exercise and better health have higher body temperature compared to rats born with low exercise capacity and disease risk. Voluntary running allowed HCRs to maintain high body temperature during aging, which suggests that high PA level was crucial in maintaining the high body temperature of HCRs.

## Introduction

Thermogenesis is an energy demanding process that has a significant contribution to daily total energy expenditure (TEE), body weight and health (Levine et al., 1999; Rosenbaum et al., 2008). High thermogenesis is linked to high physical activity (PA) level, as body temperature rises as a result of increased muscular activity (Gleeson, 1998). In rodents it has been established, that high PA in turn is tightly associated with high aerobic capacity (Novak et al., 2010). Whether the level of thermogenesis is due to inborn aerobic capacity or a consequence of PA level *per-se* remains unclear.

Several features affect thermogenesis such as age, physical activity, meals (processing nutrients), stress, and in females the stage of estrous cycle (Landsberg et al., 1984; Horan et al., 1988; Kent et al., 1991; Yamashita et al., 1994; Kontani et al., 2002; Waters et al., 2010). Physical activity, whether it is endurance or strength training, or just normal daily activities, correlates positively with body temperature (Nozu et al., 1992; Tonkonogi et al., 2000). Thermic effect of food is known to increase energy expenditure and thermogenesis (Rothwell and Stock, 1979; Cannon and Nedergaard, 2004), whereas stress causes an increase both in blood glucose concentration and body temperature via stress hormones and stress related behavior (Blanchard et al., 2001; Vachon and Moreau, 2001). In females, the stage of estrous cycle is known to affect the body temperature; temperature increases during proestrus, when progesterone and estrogen concentrations are highest, while during estrus there is a drop in body temperature (Marrone et al., 1976; Kent et al., 1991).

At cellular level, the amount and efficiency of mitochondria and uncoupling proteins (UCP) play the main role in thermogenesis (Cannon and Nedergaard, 2004; Rousset et al., 2004). UCP1 is primarily found in the mitochondria of brown adipose tissue (BAT), whereas UCP3 is the main uncoupling protein expressed in skeletal muscle (Rousset et al., 2004). UCP2 is a mitochondrial uncoupling protein that separates oxidative phosphorylation from ATP synthesis with energy dissipated as heat. Although UCP3 and UCP2 share similar features, UCP2 is mainly thought to control the production of mitochondria-derived reactive oxygen species, whereas UCP3 plays a main role in non-shivering thermogenesis (Boss et al., 1997; Rousset et al., 2004). Besides UCPs, also amount and efficiency of mitochondria to produce heat affects body temperature. PGC-1α plays a key role in mitochondrial biogenesis whereas cytochrome c (cyt c) is a crucial component of the electron transport chain in mitochondria and hence these proteins are essential for muscle oxidative capacity (Huttemann et al., 2011; Lin et al., 2015). Endurance exercise is known to increase PGC-1α levels and mitochondrial biogenesis in muscle, although UCP levels are found to be reduced (Nozu et al., 1992; Freyssenet et al., 1996; Pilegaard et al., 2000; Jones et al., 2003; Russell et al., 2003; Holloszy, 2008). Aging in general causes decrease in thermogenesis via lower UCP levels, reduced response to noradrenaline and diminished BAT content (Horan et al., 1988; Yamashita et al., 1994; Kontani et al., 2002).

BAT has for long been considered as the main thermogenic organ (van den Berg et al., 2011). At present, in addition to BAT, skeletal muscle has been demonstrated to significantly contribute to thermogenesis (Gavini et al., 2014). Since approximately 40% of total body mass is composed of muscle, the capacity of skeletal muscle to contribute to whole-body energy expenditure is reasonable. The contribution of skeletal muscle mitochondrial uncoupling to weight gain has been demonstrated in several mouse models (Son et al., 2004; Choi et al., 2007; Costford et al., 2008). It has been shown that UCP3 overexpression protects mice from high-fat diet induced insulin resistance and obesity (Clapham et al., 2000; Choi et al., 2007). Recent studies from genetically contrasting rat lines reveal that intrinsic aerobic capacity has a major role in TEE, physical activity level and non-exercise activity thermogenesis (NEAT; Novak and Levine, 2007; Gavini et al., 2014). Rats selectively bred for high aerobic endurance capacity (HCR = high-capacity runner) are also spontaneously more active and consume more energy compared to their counterparts that are selectively bred for low aerobic endurance capacity (LCR = low-capacity runner; Koch and Britton, 2001; Kivelä et al., 2010). Furthermore, HCRs have lower risk to become obese and have longer lifespan compared to LCRs, whereas LCRs are prone to gain excess body weight and develop metabolic disorders (Wisloff et al., 2005; Noland et al., 2007; Kivelä et al., 2010; Koch et al., 2011).

Body temperature is tightly associated with metabolic rate (Geiser, 1988; Heikens et al., 2011; Landsberg, 2012); in fact 1°C rise in temperature is associated with a 10–13% increment in oxygen consumption (Landsberg, 2012). The elevation in temperature itself is responsible for speeding up metabolism, since enzyme-catalyzed reactions are enhanced in higher temperatures (Landsberg et al., 2009). We recently noticed that besides the differences between the HCR and LCR rat lines listed above, anesthetized HCRs seemed to have higher body temperature compared to LCRs (*unpublished observation*). We wanted to study this observaton more carefully and measured the body temperature of HCR and LCR rats and studied the effects of aging and voluntary running for 1-year. We also followed the body temperature and spontaneous activity during glucose tolerance and placebo test to assess the effect of stress (injection) and high blood glucose. Our first hypothesis was that HCR rats have intrinsically higher body temperature compared to LCRs due to their higher mitochondrial capacity to produce heat. Secondly, we hypothesized that aging would decrease body temperature levels due to aging related loss of mitochondrial function and decreased expression of the UCP in skeletal muscle and BAT, but the difference between the HCR/LCR rat lines would still be evident. Thirdly, we assumed that voluntary running would increase the body temperature in both rat lines due to increased mitochondrial biogenesis, thus aiding to prevent age related decrease in mitochondrial function. Our final hypothesis was that glucose injection would increase the body temperature especially in the energy-dissipating HCRs, since BAT as insulin sensitive tissue may have a major role in dissipating extra energy as heat (Cannon and Nedergaard, 2004; Stanford et al., 2013).

## Materials and methods

### Animal lines

**Figure 1:**
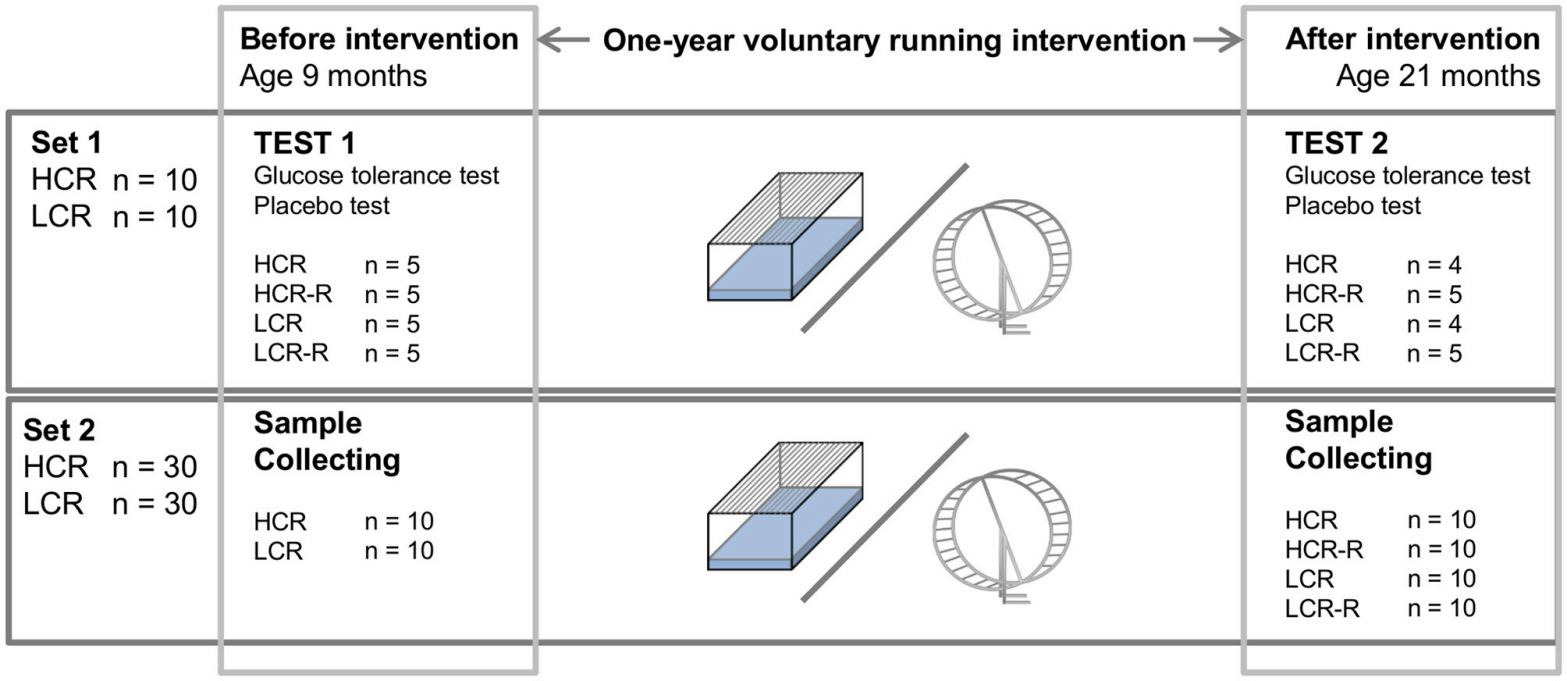
**Schematic representation of the study protocol**. There were two sets of rats with the same 1-year intervention with 4 sub-groups: HCR (control), HCR-R (runner), LCR (control), and LCR-R (runner). For set1 (20 rats) glucose tolerance and placebo tests were performed before the intervention (age 9 months) and after the intervention (age 21 months). From set 2 (60 rats) gastrocnemius muscle and brown fat samples were collected from the same time points.

The HCR/LCR contrasting rat model was produced via two-way artificial selection, starting from a founder population of 186 genetically heterogeneous rats (N:NIH stock), as described previously (Koch and Britton, 2001). Endurance running capacity was assessed at the University of Michigan (Ann Arbor, Michigan, USA) with a speed-ramped treadmill running test (15° slope, initial velocity of 10 m•min^−1^, increased 1 m/min every 2 min) when the rats were 11 weeks of age. For the glucose and placebo tests described here, 20 female rats (10 HCR and 10 LCR) from the 27th generation of selection were used (Figure 1, Set 1). First non-trained animals were tested before 1-year voluntary intervention (age 9 months) and the tests were repeated as follow-up after the intervention (age 21 months). We also used tissue samples collected from 60 female rats from generations 23–27 of selection given the same voluntary running intervention to prepare Western blots from gastrocnemius muscle and BAT at the same time points (Figure 1, Set 2). An additional set of 4–6 months old female HCR/LCR rats from the generation 35 were used to measure body surface temperatures. The rats lived in an environmentally controlled facility (12/12 h light-dark cycle, 22°C) and received water and standard feed (R36, Labfor, Stockholm, Sweden) *ad libitum*.

### Baseline measurements

#### Body temperature

Body temperature was assessed by measuring rectal temperature (Fluke 52 k/J Thermometer) after 2 h of fasting before dividing the rats into separate intervention groups (*n* = 10/group). Measurement was repeated in separate days and the average of two measurements was used for the statistical analyses.

#### Surface temperature from back and tail

An infrared camera (FLIR A300) with thermal sensitivity of <0.05°C was used to assess surface temperatures of backs and tails of HCR/LCR rats from the generation 35 (*n* = 9–12/group). For the measurement, each rat was lifted from the home cage to a separate plastic cage and three infrared images were taken at 5 s intervals. Using the software of the camera manufacturer (ThermaCAM Researcher Pro 2.1.) the highest surface temperature of the rat back and tail from each image was recorded and the mean values were regarded as the actual back or tail surface temperatures.

### Intervention

Before the first measurements, at the age of 9 months, the rats were housed two per cage in standard cages. After the first measurements rats were divided into groups evenly matched for body weight and maximal running capacity (*n* = 5); HCR (control), HCR-R (runner), LCR (control) and LCR-R (runner; Figure 1, Set 1). Control rats lived in a standard cage conditions and runners had an access to running wheels that were connected to a computerized recording system to follow the running distance throughout intervention. During this 1-year intervention the rats were housed one per cage. The follow-up measurements were performed at the age of 21 months.

#### Voluntary running distance, body weight, and energy intake

Voluntary running distance in the running wheels was followed throughout the 1-year intervention with a self-constructed computerized recording system (Acer Verinton 6900Pro, 32 bit processor produced by Intel, Windows XP). Total wheel laps were recorded continuously, and the total running distance per day was determined by multiplying the number of wheel rotations by the circumference of the running wheel (Ø 34.5 cm). From daily running distances an average daily running distance for every 2-week period was calculated. Body weight and energy intake of the rats were followed throughout the 1-year intervention by weighing the rats and consumed feed every second week. Energy intake was calculated as 2 week feed consumption from the energy content informed by the manufacturer (Labfor).

#### Body temperature: before and after intervention measurements

To study the effects of rat line, aging and voluntary running before and after 1-year voluntary running intervention, body temperature was measured similarly to the baseline measurements described above. Measurements were done after 5 h of fasting from the same rats as used for the intervention (*n* = 5/group). The average of the fasting (F) point measurements from each rat from glucose tolerance and placebo tests were used for the statistical analyses.

#### Glucose tolerance and placebo tests

The glucose tolerance and placebo tests were performed twice, first at the age of 9 months and second time at the age of 21 months (Figure 1). Tests were done in two parallel sets in each day, between 9.30 a.m. and 3.30 p.m. Rats from different groups were randomized to treatment sets to account the possible effect of circadian rhythm on blood glucose level. Two animals were excluded from the follow-up measurement of old rats due to aging symptoms, leaving the following group sizes for second measurements; HCR (*n* = 4), HCR-R (*n* = 5), LCR (*n* = 4) and LCR-R (*n* = 5). Rats were deprived of food for 5 h before measurements. The running wheels of the runner rats were blocked 5 h before the measurements disabling the movement of the wheel to avoid the possible acute effects of running on the measurements. Body weight was measured before each test. At the time point 0 either 2 g/kg of glucose (20% solution) or equal volume of placebo (physiological saline solution) was injected into the peritoneal cavity. Rectal temperature (Fluke 52 k/J Thermometer) and blood glucose were measured at time points 0, 30, 60, and 120 min after the injection. Blood samples for further analyses were collected from saphenous vein before starting the glucose/placebo protocol (fasting sample).

##### Heat accumulation

Rectal temperature was measured at time points 0, 30, 60, and 120 min after the injection. To compare the changes in temperature during placebo and glucose tolerance tests the area under curve (AUC) values of rectal temperature were calculated from each group during both tests between time points 0–120 min normalized with 0 min level. AUC values were used for the statistical analyses of the effect of rat line, running and treatment on heat accumulation.

##### Spontaneous activity

Spontaneous activity of the rats was followed throughout the glucose tolerance and placebo tests with ground reaction force recording as described before (Silvennoinen et al., 2014). The rat cages were placed on top of individual ground reaction force plates 30 min before the start of the experiments and removed from the plate after the last blood glucose measurement. The absolute values of the differences between consecutive force values were calculated. The mean of the absolute values were calculated from every second from total 20 values per second. To obtain activity index, a single value for total spontaneous activity, the one second means were summed for the total measurement time and the sum was divided by the body mass (kg) of the measured rat (Biesiadecki et al., 1999; Silvennoinen et al., 2014). The data used in statistical analyses were presented as 30 min averages. The total spontaneous activity was also calculated during the tests as a sum of activity index from the whole test period.

##### Blood glucose concentration

Blood glucose was measured at time points 0, 30, 60, and 120 min after the injection (HemoCue Glucose 201 RT). AUC normalized with 0 min concentration from the placebo and glucose tests were used for the statistical analyses of the effect of rat line, running and treatment on blood glucose concentration.

##### Phase of estrous cycle

Phase of estrous cycle was followed 1 week during the glucose tolerance and placebo measurements after the intervention (age 21 months). Estrous cycle samples were collected as epithelium samples with physiological saline solution from vagina to a pipette, stained with methylene blue (Giemsas azur-eosin; methylenblaulösung, Merck 9204) and observed under a light microscope. Each sample was categorized to one of the four menstrual cycle states: proestrus, estrus, diestrus, or metestrus.

#### Tissue processing

Gastrocnemius muscle (*n* = 10/group) and BAT (*n* = 5/group) samples were collected from the Set 2 rats (Figure 1). The snap frozen samples were homogenized in liquid nitrogen and dissolved in ice-cold buffer (20 mM HEPES (pH 7.4), 1 mM EDTA, 5 mM EGTA, 10 mM MgCl_2_, 100 mM, β-glycerophosphate, 1 mM Na_3_VO_4_, 2 mM DTT, 1% NP-40, 0.2% sodium deoxycholate, and 3% protease and phosphatase inhibitor coctail (P 78443; Pierce, Rockford, IL). The muscle homogenate was thereafter centrifuged at 10 000 g for 10 min at 4°C. Total protein content was determined using the bicinchoninic acid protein assay (Pierce Biotechnology, Rockford, IL) with an automated KoneLab instrument (Thermo Scientific, Vantaa, Finland).

##### Citrate synthase activity

Citrate synthase activity (U•μg^−1^•min^−1^) in the gastrocnemius muscle (*n* = 10/group) was measured from the same muscle homogenates that were used to determine the total protein content (Citrate Synthase Assay Kit Sigma-Aldrich) with an automated KoneLab instrument (Thermo Scientific).

##### Western blot analyses

Aliquots of muscle homogenate were solubilized in Laemmli sample buffer and heated at 95°C to denaturate proteins, except for Total OXPHOS Cocktail, when samples were heated at 50°C. Thereafter, samples containing 30 μg of total protein were separated by SDS-PAGE for 60 to 90 min at 200 V using 4–20% gradient gels on Criterion electrophoresis cell (Bio-Rad Laboratories, Richmond, CA). Proteins were transferred to PVDF membranes at 300 mA constant current for 2 h on ice at 4°C. The homogeneity of protein loading was checked by staining the membrane with Ponceau S. Membranes were blocked in TBS with 0.1% Tween 20 (TBS-T) containing 5% non-fat dry milk for 2 h and then incubated overnight at 4°C with commercially available polyclonal primary phosphospecific antibodies to measure the following protein contents with stated dilutions: GAPDH (1:10000; ab9485, Abcam), tubulin (1:1500; T6199, Sigma), PGC-1α (1:4000; 516557, Calbiochem), cyt c (1:500; sc-8385, Santa Cruz biotechnology, Inc.), UCP2 (1:300; ab67241 Abcam), UCP3 (1:3000; ab3477 Abcam), and Total OXPHOS Cocktail (1:1000; ab110413; Abcam). All the antibodies were diluted in TBS-T containing 2.5% non-fat dry milk.

BAT samples were treated as above and prepared for Western-blot analyses for PGC-1α, cyt c, UCP1 (1:4000; ab10983, Abcam), UCP2, and UCP3.

After the primary antibody incubation membranes were washed in TBS-T, incubated with suitable secondary antibody diluted in TBS-T with 2.5% milk for 1 h followed by washing in TBS-T. Proteins were visualized by ECL according to the manufacturer's protocol (SuperSignal West femto maximum sensitivity substrate, Pierce Biotechnology) and quantified using ChemiDoc XRS in combination with Quantity One software (version 4.6.3. Bio-Rad Laboratories). The UCP3 and pACC membranes described above were incubated in Restore Western blot stripping buffer (Pierce Biotechnology) for 30 min and reprobed with GAPDH or total-ACC antibodies by immunoblot analysis as described above. Results from gastrocnemius muscle were normalized to the corresponding level of GAPDH (PGC-1α, cyt c and UCP3), tubulin (UCP2, total-ACC and pACC), or PonceauS stained actin band (OXPHOS Cocktail). All proteins from BAT samples were normalized to corresponding level of tubulin.

#### Serum cortisol concentration

Cortisol concentration was measured from frozen (−80°C) serum samples by a kinetic photometric method with KoneLab (Thermo Scientific).

#### Ethics statement

This study was approved by the National Animal Experiment Board, Finland (Permit number ESAVI-2010-07989/Ym-23).

### Statistical analyses

All values in figures are expressed as mean ± standard error of the mean (SEM). Statistical analyses for variables were carried out using SPSS for Windows 22 statistical software (version 22, IBM SPSS Statistics) and in Mplus 7. The Shapiro-Wilk test was used to investigate within group normality for a given parameter of interest. Levene's test was conducted to assess the homogeneity of variance assumption. Univariate analysis was done to analyze the line, age, running and treatment effects to the measured parameters with Tukey *Post-hoc* test. Body surface temperatures were analyzed using *T*-test. When the normality or equality of variance assumptions were not met, statistical comparisons of parameters between LCR and HCR groups were made using Mann–Whitney test. The comparison between before and after intervention parameters within the same group were done using Wilcoxon test. Mixed model analysis controlled for age, running (yes/no), treatment (placebo/glucose), time point (F, 0, 30, 60, and 120 min), and spontaneous activity, was used to determine the effect of line (HCR or LCR) on the measured body temperature levels. Separate analysis for both rat lines of the effect of running, treatment, time point, and spontaneous activity on rectal temperature were performed using 4 × 5 longitudinal covariance structure analyses in Mplus 7 for both glucose tolerance and placebo tests. *P*-values less than 0.05 were considered statistically significant.

## Results

### Baseline measurements

#### Body temperature

**Figure 2:**
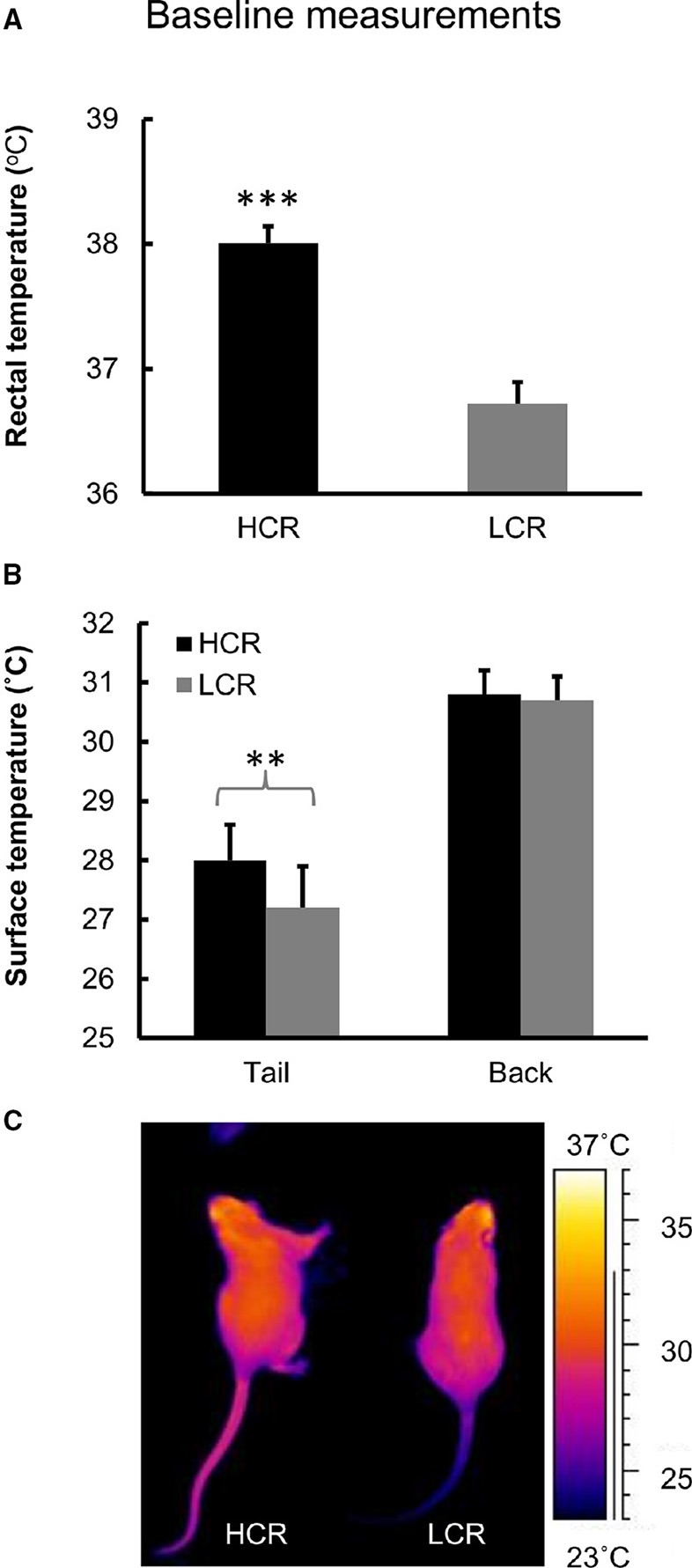
**Baseline measurements**. **(A)** Baseline measurement of body temperature. *n* = 10/group, ^***^p < 0.001. **(B)** Baseline measurement of body surface temperature from tail and back. *n* = 9–12/group, ^**^p < 0.010. Values are expressed as mean ± SEM. **(C)** Infrared image of HCR and LCR rat.

HCRs had on average 1.3°C higher body temperature after 2 h of fasting compared to LCRs (*p* < 0.001; Figure 2A).

#### Surface temperature from back and tail

HCRs had higher surface temperature from tail compared to LCRs (*p* < 0.01; Figures 2B,C). There was no difference in the surface temperatures measured from the back (Figures 2B,C).

### Intervention measurements

#### Before and after 1-year running intervention measurements

**Figure 3:**
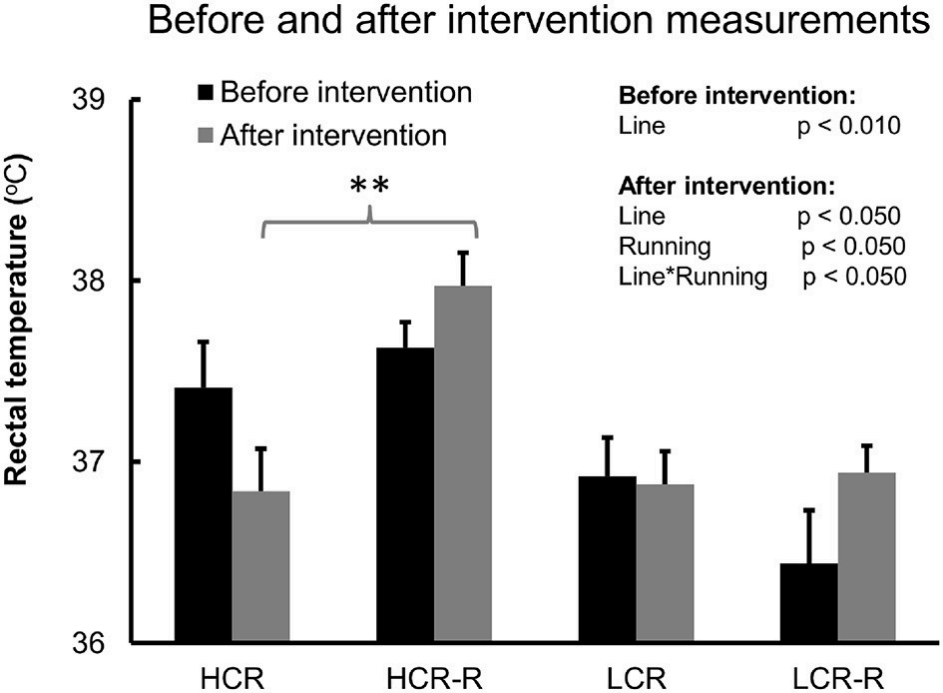
**Before and after intervention measurements of body temperature**. Before and after intervention measurements of body temperature. *n* = 4–5/group, ^**^p < 0.010 *post-hoc* test between HCR and HCR-R. Univariate analysis of rat line and running effect before and after 1-year intervention. Values are expressed as mean ± SEM.

Univariate analysis showed a significant line effect on body temperature both before and after intervention (*p* < 0.050), with HCRs having higher body temperature compared to LCRs (Figure 3). When only control groups (HCR vs. LCR) were included in the analyses, the line effect after intervention diminished. Aging did not have a marked impact on body temperature, whereas running had a significant effect after intervention (*p* < 0.050) showing an increase in the body temperature levels in both rat lines (*p* < 0.050, Figure 3). Also combined effect of line and running was significant after intervention (*p* < 0.050). *Post-hoc* test showed that after the intervention, HCRs in the runner group had higher body temperature compared to the corresponding controls (*p* < 0.010), whereas there was no difference between the LCR groups (Figure 3).

#### Voluntary running distance, body mass, and energy intake

**Figure 4:**
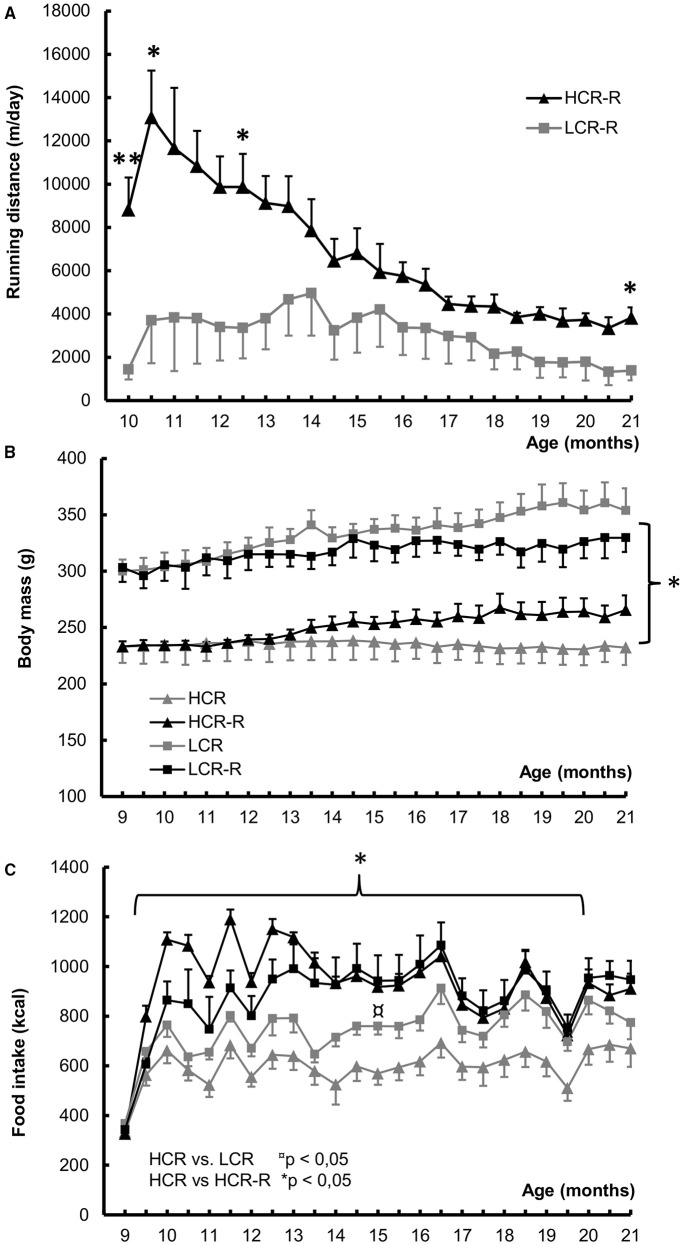
**Voluntary running distance, Body mass and Food intake during 1-year intervention**. **(A)** Voluntary running distance (m/day). HCRs had longer voluntary running distance than LCRs, the difference being significant at time points 10, 10.5, 12.5, and 21 months of age, ^*^p < 0.050, ^**^p < 0.010. **(B)** Body mass (g). LCR rats in both groups were heavier compared to HCR rat groups during the follow-up period (^*^p < 0.050). In LCRs the rats in the runner group were leaner than control ones, whereas the opposite was true for HCRs. There were no statistical differences within the rat lines. **(C)** Food intake (kcal). LCRs in the control groups had higher food intake compared to corresponding HCRs, the difference was significant at time points 15 and 19.5 months of age (*p* < 0.050). Runner groups of both rat lines consumed more energy compared to the corresponding control groups, but only in HCR line this difference was significant (HCR vs. HCR-R, *p* < 0.050). *n* = 4–5/group, values are expressed as mean ± SEM.

HCRs ran voluntarily more than LCRs, the difference being significant at time points 10, 10.5, 12.5, and 21 months of age (*p* < 0.05; Figure 4A). When comparing the body weight, LCR rats in both groups were heavier than HCR rats during the whole follow-up period (*p* < 0.05; Figure 4B). There was no statistical difference within the rat lines. LCRs in the control groups had higher food intake compared to corresponding HCRs, the difference being significant at time points 15 and 19.5 months of age (*p* < 0.05). Both runner groups consumed more energy compared to the corresponding control groups (Figure 4C), but only in HCR line this difference was significant (HCR vs. HCR-R, *p* < 0.05).

#### Glucose tolerance and placebo tests

##### Heat accumulation

**Figure 5:**
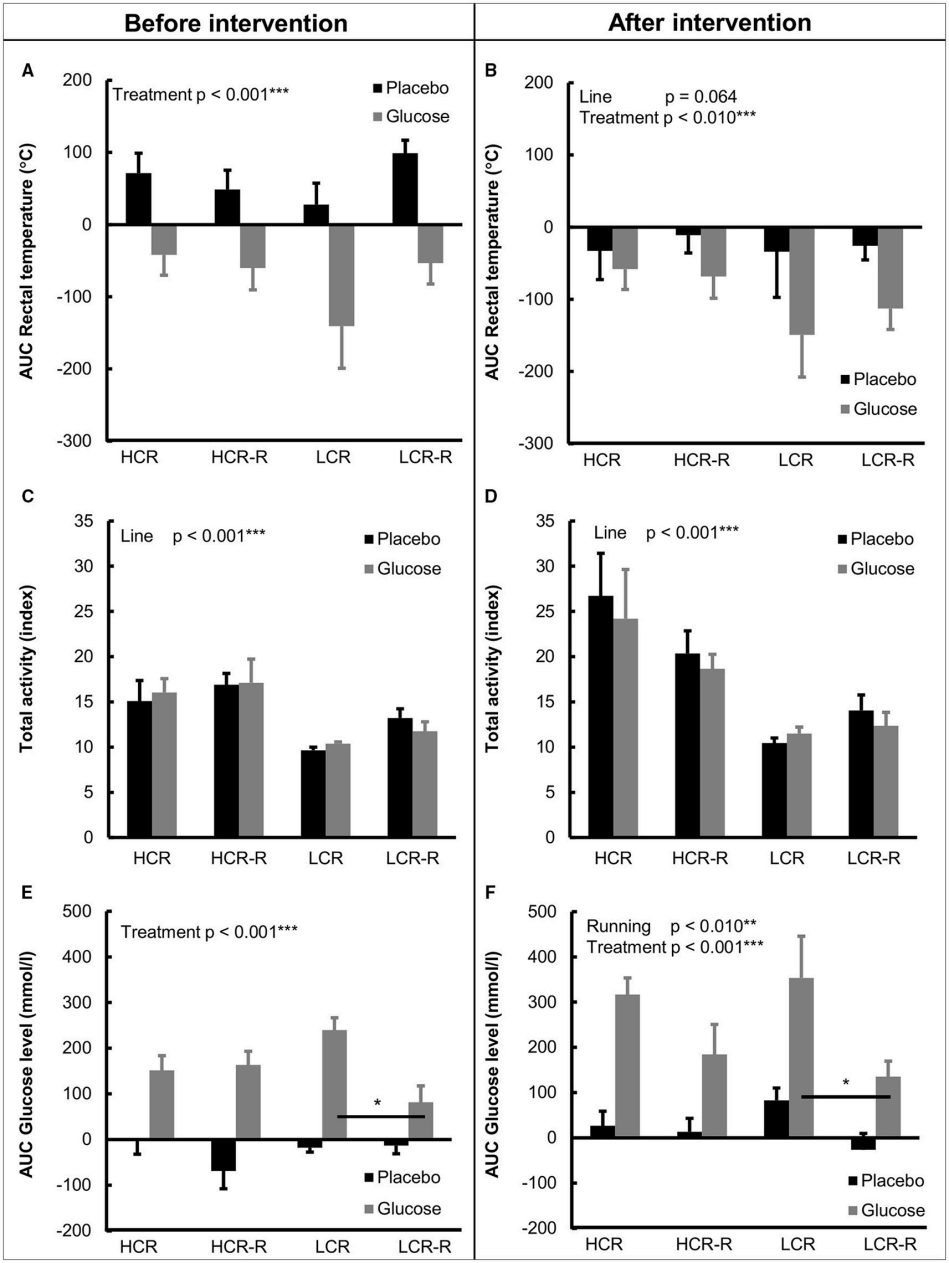
**Heat accumalation (AUC rectal temperature), total activity and blood glucose concentration (AUC) during placebo and glucose tolerance tests**. **(A,B)** AUC Rectal temperature. Before the 1-year intervention treatment had a significant effect on heat accumulation (*p* < 0.001), with glucose injection lowering the heat accumulation. After the intervention treatment was still significant (*p* < 0.010) and line effect was nearly significant (*p* = 0.064). **(C,D)** Total activity. HCRs had higher total activity compared to LCRs both before and after 1-year intervention (line effect *p* < 0.001). **(E,F)** AUC Glucose concentration. Treatment had a significant contribution to blood glucose AUC concentration both before and after intervention (*p* < 0.001), with glucose injection increasing blood glucose AUC. *Post-hoc* test revealed that LCRs controls had higher blood glucose AUC compared to the corresponding runners both before and after the intervention (^*^p < 0.050). Univariate analysis of effects of rat line, treatment (glucose/placebo injection) and voluntary running on studied parameters. *n* = 4–5/group, values are expressed as mean ± SEM.

Before the intervention, heat accumulation was positive when normalized to 0-value during placebo and negative during glucose test, and LCR control group had the largest negative response to glucose injection (Figure 5A). Univariate analysis showed an effect of treatment on heat accumulation (*p* < 0.001, Figure 5A). After the intervention treatment still had a significant effect on heat accumulation (*p* < 0.010, Figure 5B) and the effect of line was nearly significant (*p* = 0.064). Responses to both placebo and glucose injections in all groups were negative after the intervention, with both LCR groups having greater negative response to glucose injection compared to HCR groups (Figure 5B).

##### Spontaneous activity

Total activity during the test protocols are presented in Figures 5C,D. Univariate analysis revealed that HCRs had higher total activity during placebo and glucose tolerance tests both before and after the 1-year voluntary running intervention compared to LCRs (line effect *p* < 0.001, Figures 5C,D).

##### Blood glucose concentration

Before the intervention the blood glucose AUC value in all groups was negative during placebo test and positive during glucose tolerance test (Figure 5E). Univariate analysis showed significant treatment effects both before and after the intervention (*p* < 0.050, Figures 5E,F). *Post-hoc* test further revealed that LCRs in the control group had higher blood glucose AUC concentration compared to the corresponding runners both before and after the intervention (*p* < 0.050), whereas there was no statistical difference between the HCR groups.

#### Serum cortisol concentration

**Figure 6:**
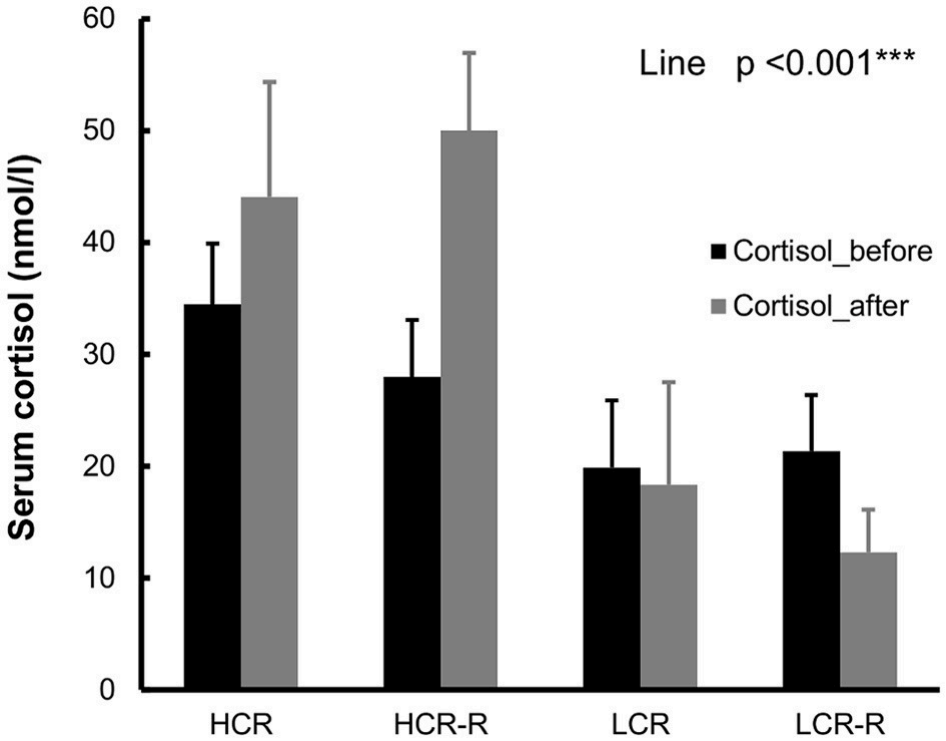
**Cortisol**. HCRs had higher cortisol concentrations compared to LCRs both before and after intervention (line effect *p* < 0.001). Serum analyses were done from the fasting blood samples collected before and after intervention. *n* = 4–5/group, values are expressed as mean ± SEM.

HCRs had higher cortisol concentration compared to LCRs both before and after intervention (line effect, *p* < 0.001, Figure 6).

### Western-blot analyses

#### Gastrocnemius muscle

**Figure 7:**
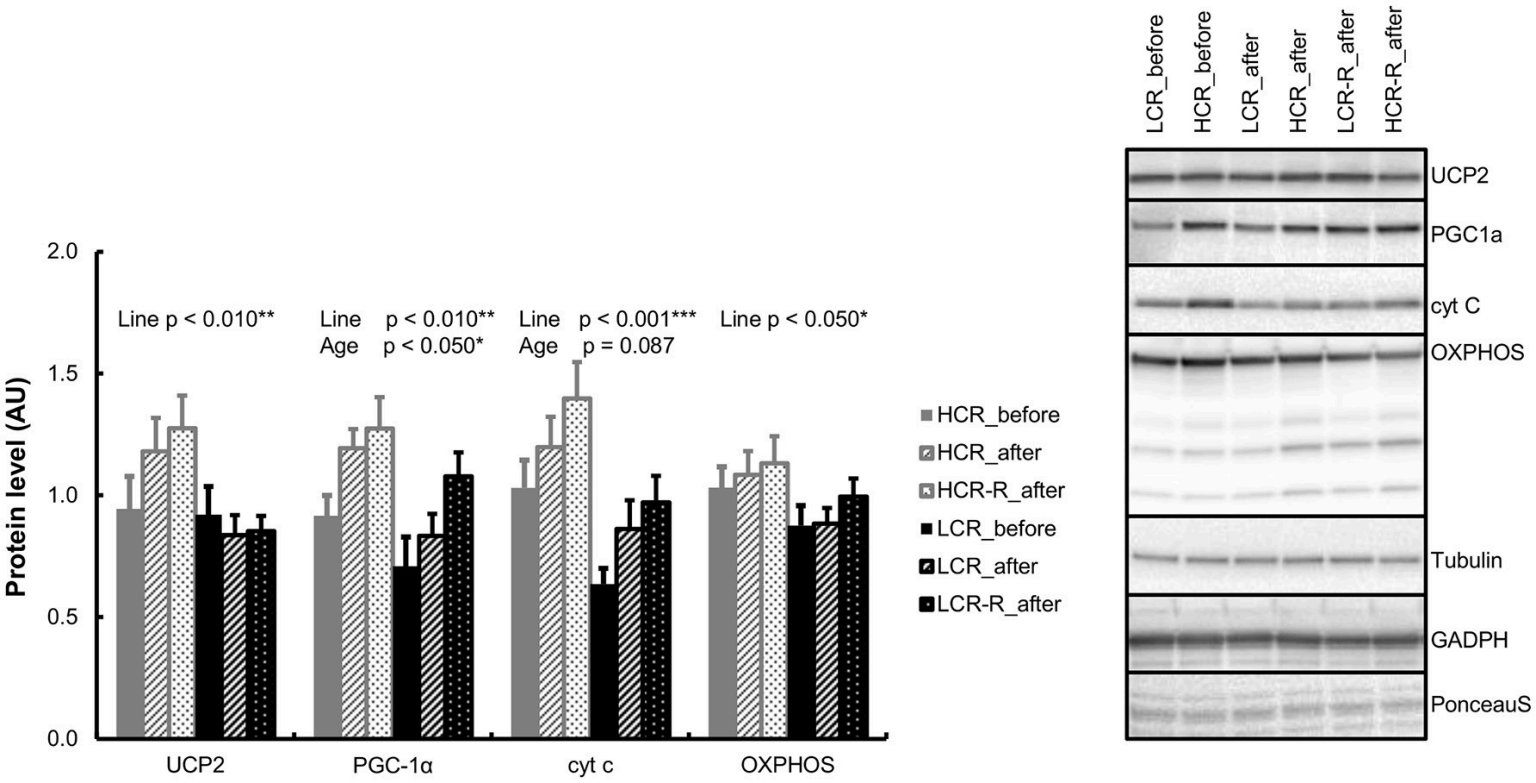
**Western blot analyses from gastrocnemius muscle**. HCRs had higher UCP2, PGC-1α, cyt c, and OXPHOS protein levels (line effect *p* < 0.050). Aging increased the level of PGC-1α (*p* < 0.050) and there was a tendency in increase of cyt c level with aging (*p* = 0.087). *n* = 9–10/group, values are expressed as mean ± SEM.

Univariate analysis showed a significant line effect in UCP2 and OXPHOS (*p* < 0.050) with HCRs having higher protein levels compared to LCRs (Figure 7). HCRs had also higher PGC-1α and cyt c protein levels than LCRs (*p* < 0.050, *reported previously*; Figure 7). Aging also increased the level of PGC-1α (*p* < 0.050) and there was a tendency in increase of cyt c level with aging (*p* = 0.087; *reported previously*). There were no statistical differences in the other studied protein contents.

#### BAT

Line, running or aging had no significant effect on BAT protein contents (*data not shown*).

### Citrate synthase activity

HCRs had higher citrate synthase activity compared to LCRs (line effect *p* < 0.05, *reported previously*). The activity levels were following: Before intervention: HCR 2939 ± 679, LCR 2767 ± 866 and after intervention: HCR 3267 ± 1081, HCR-R 3554 ± 835, LCR 2863 ± 917 and LCR-R 3545 ± 1328 (U•μg^−1^•min^−1^, mean ± *SD*). Aging or voluntary running had no significant effect on citrate synthase activity.

### Body mass and relative gastrocnemius muscle and bat masses

Body and relative masses of gastrocnemius muscle and BAT are listed in Table 1. Before the intervention, HCRs had lower body mass and higher relative gastrocnemius muscle mass compared to LCRs (line effect *p* < 0.001). Aging had a significant effect in both body mass and relative gastrocnemius muscle mass (*p* < 0.050); before the intervention HCRs had higher relative gastrocnemius muscle mass compared to HCRs after the intervention, while LCRs before intervention had higher relative gastrocnemius mass compared to both LCR and LCR-R after intervention. Line, running or age had no significant effect on relative BAT mass.

**Table 1:** **Background information for Western blot analyses**.

	**Body mass (g)**	**Gastrocnemius/Body mass (mg/g)**	**Brown fat/Body mass (mg/g)**
HCR_young	232 ± 30	5.32 ± 0.65	
HCR_old	260 ± 36	4.57 ± 0.58	1.33 ± 0.22
HCR-R old	268 ± 34	4.78 ± 0.49	1.23 ± 0.27
LCR_young	302 ± 26	4.73 ± 0.43	
LCR_old	320 ± 37	4.21 ± 0.45	1.02 ± 0.36
LCR-R_old	345 ± 48	4.00 ± 0.59	1.16 ± 0.26
*p*	Line < 0.001^***^	Line < 0.001^***^	
	Age < 0.050^*^	Age < 0.001^***^	

*Body and relative tissue masses (tissue mass/body mass) of the rats used for western blot analyses. n = 10/group, values are expressed as mean ± SD*.

### Estrous cycle

There were no statistical differences between the groups in the observed stages of estrous cycle during glucose tolerance and placebo tests (Table 2).

**Table 2:** **Stage of estrous cycle**.

**N**	**HCR**		**HCR-R**		**LCR**		**LCR-R**	
	**Placebo**	**Glucose**	**Placebo**	**Glucose**	**Placebo**	**Glucose**	**Placebo**	**Glucose**
**STAGE OF ESTROUS CYCLE**								
Proestrus	1	1	0	0	1	1	1	2
Estrus	0	0	0	3	1	2	1	2
Metestrus	2	1	2	1	1	1	2	1
Diestrus	1	2	3	1	1	0	1	0

*Estimated stage of estrous during the second (age 21 months) glucose tolerance and placebo tests. Numbers are n of animals that are in the presented stage of estrous cycle. There were no statistical differences between the groups*.

### Mixed model and longitudinal covariance structure analysis of body temperature

Mixed model analyses from intervention measurements revealed, that rat line had clear impact on body temperature both before and after intervention (*p* < 0.01, Table 3). Longitudinal covariance analysis showed that before the 1-year voluntary running intervention the spontaneous activity level and protocol time point (F, 0, 30, 60, or 120 min) had the largest effect on body temperature of HCRs (*p* < 0.05, Table 3), whereas in LCRs, treatment (placebo/glucose injection) and spontaneous activity had highest impact on body temperature (*p* < 0.001). After the intervention both running and spontaneous activity had a significant contribution to the body temperature levels of HCRs (*p* < 0.05). In LCRs treatment and time point had marked impact on body temperature after the intervention (*p* < 0.05).

**Table 3:** **Body temperature during test protocols: effect of measured parameters**.

		**Factor**	***p***
Before intervention		Line	< 0.000^***^
	HCR	Running	0.510
		Treatment	0.067
		Time point	0.036^*^
		Spontaneous activity	0.037^*^
	LCR	Running	0.955
		Treatment	< 0.001^***^
		Time point	0.402
		Spontaneous activity	< 0.001^***^
After intervention		Line	< 0.010^**^
	HCR	Running	< 0.001^***^
		Treatment	0.815
		Time point	0.387
		Spontaneous activity	0.014^*^
	LCR	Running	0.330
		Treatment	0.013^*^
		Time point	0.010^*^
		Spontaneous activity	0.370

*Mixed model and longitudinal covariance structure analysis of how measured parameters affect absolute body temperature levels during placebo and glucose tolerance tests. Parameters: running (yes/no), treatment (glucose/placebo injection), time point (F, 0, 30, 60, or 120 min), and spontaneous activity before and after voluntary running intervention. n = 4–5 /group*.

* *p < 0.050*,

** *p < 0.010*,

*** *p < 0.001*.

## Discussion

In the present study we examined the association of intrinsic aerobic capacity, aging, voluntary running and blood glucose concentration on body temperature in two genetically contrasting rat lines (HCR/LCR) that widely differ for their intrinsic (i.e., non-trained) aerobic capacity (Koch and Britton, 2001). Our findings show that at young age, in untrained state, HCRs have higher body temperature than LCRs. We also found that voluntary running aided HCRs to maintain high body temperature during aging. Glucose injection lowered the body temperature of LCRs, whereas no significant effect of blood glucose on body temperature was found in HCRs.

As hypothesized, our study showed that untrained HCRs had on average 1.3°C higher body temperature than corresponding LCRs (Figure 2A). There was also an apparent rat line effect when measuring the baseline body temperatures before 1-year voluntary running intervention (Figure 2B). Since normal body temperature range in rats is 35.9–37.5°C (Animal care and use committee, John Hopkins University, Baltimore, Maryland, USA), HCRs seem to have slightly elevated body temperature compared to reference values. Previous findings have shown that female HCRs have higher muscle heat dissipation during activity, explaining their higher total energy expenditure compared to LCRs (Gavini et al., 2014). However, in that study critical factor was activity related thermogenesis, whereas no significant contribution of resting metabolic rate was found when body size and composition were considered (Gavini et al., 2014). As shown in previous study by Novak et al. and here, HCRs are spontaneously more active than LCRs (Figures 5C,D) and activity level also contributed to body temperature level (Table 3), making spontaneous activity a potential cause for the higher thermogenesis of HCRs (Novak et al., 2010). However, the total activity of the rats was very similar during both placebo and glucose tolerance test showing no effect of treatment on activity level (Figures 5C,D). Yet there was a clear difference in the heat accumulation between the two protocols (Figures 4A,B), indicating that the higher heat accumulation of HCRs is not solely explained by their PA level. Although the back surface temperatures of HCRs and LCRs did not differ, HCRs had significantly higher tail surface temperature (Figures 2B,C). Since rats use their tail blood circulation for thermoregulation (Raman et al., 1983), HCRs seem to dissipate more heat suggesting that HCRs also have higher thermogenesis than LCRs. This is also supported by our previous results showing that HCRs have higher resting metabolic rate than LCRs (Kivelä et al., 2010).

In the present study HCRs had higher UCP2, PGC-1α, cyt c, and OXPHOS levels compared to LCRs, showing a greater oxidative phosphorylation capacity (Figure 7). Interestingly, a previous study has shown that overexpression of PGC-1α in skeletal muscle leads to elevated proton leak i.e., less efficient mitochondrial respiration (St-Pierre et al., 2003). Since HCRs have more OXPHOS proteins and higher UCP2 and PGC-1α levels in skeletal muscle, it may be speculated that the higher proton leakage may also be one reason for HCRs higher heat production. On the other hand, several animal models have demonstrated that rodents with inherited obesity have low body temperatures (Trayhurn et al., 1977; Levin et al., 1981; Dubuc et al., 1985). Here we show for the first time, that similarly to inherited obesity, LCRs have lower body temperature than HCRs, and are prone to gain excess weight and develop metabolic disorders (Koch and Britton, 2005; Noland et al., 2007; Kivelä et al., 2010).

Contrary to our second hypothesis, aging *per-se* had no significant effect on body temperature (Figure 3). This is in line with a previous study, where aging did not have an impact on rectal temperatures (3–24 months old), until in later age (36 months; Horan et al., 1988; McDonald et al., 1989). However, aging increased the level of PGC-1α and had a tendency to increase cyt c level (Figure 7), not showing expected aging related decrease of mitochondrial function and content as shown previously (Conley et al., 2000; Huang and Hood, 2009; Johnson et al., 2013). Despite these unexpected findings from muscle tissue level, aging did diminish the difference in the body temperature between the rat lines when comparing only the control groups (HCR vs. LCR, Figure 2B). This was due to decrease in body temperature of HCRs; aging did not have a marked impact on the body temperature of LCRs. For an unknown reason, HCRs seem to lose their ability to keep up high heat generation with aging at control conditions (e.g., no running wheel). Our results revealed that aging significantly diminished the relative gastrocnemius muscle mass in HCRs (Table 1), which may partly contribute to the aging related lower heat generation.

As we hypothesized, 1-year voluntary running did increase the body temperature in both rat lines showing a significant running effect (Figure 3). Further analyses showed that running had a significant impact on the body temperature of HCRs (Table 3), which is consistent with their running distances compared to LCRs (Figure 4A). It has been established in previous studies, that endurance training increases PGC-1α and cyt c levels as well as mitochondrial content and respiratory capacity in skeletal muscle in both humans and rodents (Holloszy and Coyle, 1984; Booth, 1991; Baar et al., 2002; Pilegaard et al., 2003). Surprisingly, voluntary running had no significant effect on the mitochondrial proteins in the present study. It seems that in gastrocnemius muscle, there was no pressure to increase the mitochondrial proteins at time point chosen (age of 21 months). PGC-1α is known to respond to exercise acutely (Baar et al., 2002). Like in other studies, also in our study the running distance decreased gradually over time (Holloszy, 1993; Judge et al., 2005), also the pressure for change decreased (Figure 4A). Contrary to the increased body temperature by voluntary running established in our study, in previous studies endurance training has been associated with decreased mRNA expression of the uncoupling proteins in skeletal muscle and reduced thermogenesis in BAT (Nozu et al., 1992; Boss et al., 1998).

It is worth noting that lifespan is generally negatively correlated with body temperature (Rikke and Johnson, 2004). Several life-extending manipulations in rodents, such as caloric restriction, have shown to decrease body temperature by 1–5°C (Rikke and Johnson, 2004). Furthermore, a modest prolonged reduction of core body temperature (0.3–0.5°C) increased median life expectancy in mice 12–20% even without caloric restriction (Conti et al., 2006). Nonetheless, in the HCR/LCR animal model, HCRs have higher body temperature associated with longer lifespan than LCRs (Koch et al., 2012; Karvinen et al., 2015). The free radical hypothesis of aging includes ‘uncoupling to survive’ hypothesis, which suggests that correlation between metabolic rate and longevity should be positive (Brand, 2000). In a previous study, it was estimated that mitochondrial proton cycling causes up to 20–25% of basal metabolic rate in rats (Rolfe et al., 1999). It was suggested that the function of the energy-dissipating proton leak is not primarily to increase thermogenesis, but to decrease the production of reactive oxygen species. Indeed, proton leak is proposed to be a key factor in aiding to decrease oxidative damage to DNA and to slow down aging (Brand, 2000; Speakman et al., 2004). It seems that HCRs are good candidates supporting the ‘uncoupling to survive’ hypothesis since their higher OXPHOS, PGC-1α, and UCP2 levels combined with higher body temperature and longer lifespan compared to LCRs (Koch et al., 2012; Karvinen et al., 2015).

Previous studies have also shown that core body temperature declines with age both in rodents and in humans (Roth et al., 2002; Sanchez-Alavez et al., 2011; Waalen and Buxbaum, 2011), which has raised speculation of possible anti-aging effects of low body temperature. In our studies, HCRs in the control group had lower body temperature at the age of 21 months than HCR-Rs, and our previous study reported that HCRs in control group had also longer lifespan (Karvinen et al., 2015). According to our results it can be speculated that voluntary running may interfere with a natural aging-related reduction in body temperature of HCRs, possibly contributing to a decrease in lifespan. However, it remains controversial whether high body temperature and high rate of metabolism are beneficial to health and longevity, and more studies are needed to investigate the role of metabolic rate on aging and longevity.

Our final hypothesis was that glucose injection would increase the body temperature especially in HCRs, increasing heat generation in BAT. Our results revealed, that especially in LCRs, heat accumulation was lower after glucose tolerance test compared to placebo test (Figures 5A,B). Longitudinal covariance analysis further revealed that treatment was a significant contributor to temperature level in LCRs both before and after intervention (Table 3). It may be speculated, that in LCRs glucose injection activates energy storage mechanisms that decrease their metabolic rate shortly after energy supplementation (Ravussin and Gautier, 1999; Almind and Kahn, 2004; Landsberg et al., 2009; Heikens et al., 2011). However, it should be noticed that there was a difference in the blood glucose AUC level between the LCR groups already before the 1-year running intervention started (Figure 5C), which is most likely due to large variation in the response to glucose dose due to small group size. After the intervention rats in the running groups had a lower response to glucose; however, again the *post-hoc* tests revealed that the difference was only significant between the LCR groups (Figure 5E).

In addition to aging and voluntary running, other potential factors may affect the body temperature in our setup, e.g., handling of the animal and estrous cycle. Handling is known to have a stress effect on rats and it can cause a series of behavioral and physiological responses, such as flight response, freezing response, increase in heart rate, urination and increase in plasma glucocorticoid levels (Rodgers et al., 1997; Blanchard et al., 2001). It is known that HCRs have stronger response to stress, and this can be observed also in elevated corticosterone level in blood in HCRs compared to LCRs (Waters et al., 2010). Similarly in our study HCRs had higher cortisol concentration compared to LCRs the (Figure 6). This stronger response to stress can also be one reason for the higher body temperature of HCRs (Vachon and Moreau, 2001). Estrous cycle affects the body temperature in female rats; it increases during proestrus, and drops during estrus (Marrone et al., 1976; Kent et al., 1991). In our study the stage of estrous cycle was estimated during the follow-up measurements. Rats were randomly in one of the four estrous phases, and no significant differences in the estrous cycle stages between the rat lines were observed (Table 2).

To conclude, HCRs have higher basal body temperature than LCRs at untrained state at young age. Voluntary running aids older HCRs to maintain body temperature at similar levels as untrained, younger HCRs. The elevated body temperature itself may partly cause the heightened oxidative metabolism in HCRs, as enzyme-catalyzed reactions are enhanced in higher temperatures (Landsberg et al., 2009). However, voluntary running may interfere with a natural aging-related reduction in body temperature of HCRs, possibly contributing to a decrease in lifespan (Karvinen et al., 2015). On the other hand it is proposed that low body temperature is one reason for the onset of obesity in humans (Landsberg et al., 2009). Yet it remains controversial whether high body temperature and high rate of metabolism are beneficial to health and longevity. However, since there is clear evidence of obese animals having low body temperatures (Trayhurn et al., 1977; Levin et al., 1981; Dubuc et al., 1985), it would be worth studying the potential role of intrinsically low body temperature at the onset of obesity in humans.

## Author contributions

HK, MS, and SK designed the study. HK led the animal experiment and SK performed the animal experiment and analyzed the data. HM, MS, SK, and RT collected the tissue samples. TR and MS built up the spontaneous activity measurement system. TT and SL assisted with the statistical analysis and interpretation of the data. SK, HK, and MS drafted the manuscript. SB and LK bred and phenotyped the animals. All authors contributed to the revision of the manuscript and approved the final version of the manuscript.

## Funding

This study was funded by the Finnish Ministry of Education and Culture, National Doctoral Programme of Musculoskeletal Disorders and Biomaterials (TBDP) and Eemil Aaltonen foundation. The LCR-HCR rat model system was funded by the Office of Research Infrastructure Programs/OD grant P40OD021331 (to LK and SB) from the National Institutes of Health.

### Conflict of interest statement

The authors declare that the research was conducted in the absence of any commercial or financial relationships that could be construed as a potential conflict of interest.
